# Effect of Different Titanium Surfaces on Maturation of Murine Bone Marrow-Derived Dendritic Cells

**DOI:** 10.1038/srep41945

**Published:** 2017-02-03

**Authors:** Xiaofei Zheng, Fengjuan Zhou, Yifei Gu, Xiaobo Duan, Anchun Mo

**Affiliations:** 1 State Key Laboratory of Oral Diseases, West China Hospital of Stomatology, Sichuan University, Chengdu, Sichuan, China; 2 Dental Implant Center, West China Hospital of Stomatology, Sichuan University, Chengdu, Sichuan, China; 3 Geriatric Dentistry Department, West China Hospital of Stomatology, Sichuan University, Chengdu, Sichuan, China

## Abstract

Dendritic cells (DCs) play a pivotal role in the host response to implanted biomaterials. Osseointegration of titanium (Ti) implant is an immunological and inflammatory-driven process. However, the role of DCs in this complex process is largely unknown. This study aimed to investigate the effect of different Ti surfaces on DC maturation, and evaluate its subsequent potential on osteogenic differentiation of preosteoblasts. Murine bone marrow-derived DCs were seeded on Ti disks with different surface treatments, including pretreatment (PT), sandblasted/acid-etched (SLA) and modified SLA (modSLA) surface. Compared with DCs cultured on PT and SLA surfaces, the cells seeded on modSLA surface demonstrated a more round morphology with lower expression of CD86 and MHC-II, the DC maturation markers. Those cells also secreted high levels of anti-inflammatory cytokine IL-10 and TGF-β. Notably, addition of conditioned medium (CM) from modSLA-induced DCs significantly increased the mRNA expression of Runx2 and ALP as well as ALP activity by murine preosteoblast MC3T3-E1 cells. Our data demonstrated that Ti disks with different surfaces lead to differential DCs responses. PT and SLA surfaces induce DCs mature, while DCs seeded on modSLA-Ti surface maintain an immature phenotype and exhibit a potential of promoting osteogenic differentiation of MC3T3-E1 cells.

When implanted into living tissue, all biomaterials will elicit the host response that is regulated by surface properties and predicates the first step of tissue repair^1^. Dendritic cells (DCs), the most efficient antigen presenting cells, are considered as initiator and regulator of the immunity, playing an important role in the host response to implant biomaterials^2^. The functional activities of DCs are mainly dependent on their state of maturation and the cytokine environment^3^. The terminally differentiated mature DCs (mDC) can efficiently induce immunity, whereas immature DCs (iDC) are promoters of immune tolerance^4^.

Distinct biomaterials had different influences on the phenotype of DCs^5^. Specifically, poly(lactic-co-glycolic acid) and chitosan films induced DC to be mature, while agarose films and hyaluronic acid films maintained an immature phenotype of DC^6^,^7^. These above-mentioned studies indicated that biomaterials can be used to regulate DC phenotype, thereby controlling the related *in vivo* host response to the implant biomaterials.

Titanium (Ti) dental implants have been widely used to restore missing teeth. The direct contact of implants with the bone tissue, also known as osseointegration, is the most important factor that determines the success of an implant. As to titanium implants, we expect the minimum immune response and the rapid osseointegration for the proper function. The buildup of osseointegration is complicated, which seems to be an immunological and inflammatory-driven process^8^. The knowledge of this process is essential for the optimization of Ti surface modification^9^. Therefore, the involved parts and their roles in this complex healing process should be understood, such as the cells and mediators, as well as the phenotype changes of these participant cells.

The design of modern implant aims at utilizing the favorable immune response to improve osseointegration while avoiding the chronic inflammation and foreign body reactions which would lead to the loss of the intended function^10^. Both *in vitro* and *in vivo* studies have suggested that Ti implants with different surface modifications showed different effects on the proliferation and differentiation of osteoblasts, leading to differential bone reactions. Compared with smooth pretreatment (PT) surface, Ti implants with moderate roughness can enhance the bone-implant contact^11^. The sandblasted/acid-etched (SLA) surface treatment significantly promoted new bone apposition^12^,^13^. Implants with chemically modified sandblasted/acid-etched (modSLA) treatment demonstrated a significantly greater mean percentage of bone-implant contact and higher removal torque values than those with conventional SLA surface^14^,^15^,^16^.

Once the dental implants are inserted into the bone tissue, DCs do come in contact with the implants. This study aims to evaluate the response of murine bone marrow-derived DCs to different Ti surfaces and to investigate the subsequent potential of Ti-induced DCs on the osteogenic differentiation of preosteoblast MC3T3-E1 cells.

## Results

### Ti surface characteristics

**Figure 1:**
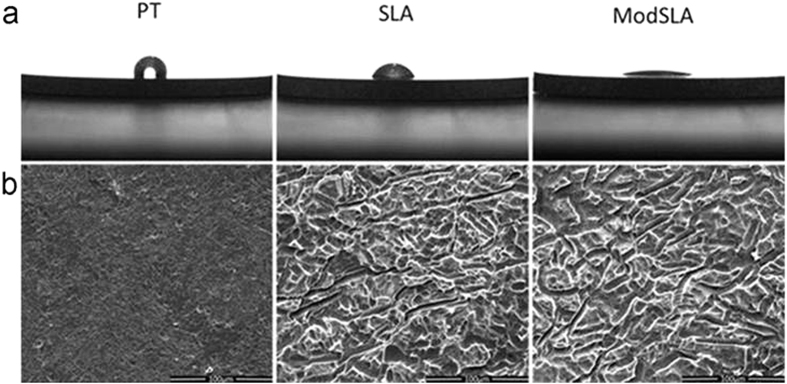
(**a**) The contact angles and (**b**) SEM micrographs (original magnification ×100, bar = 100 μm) of pretreatment (PT), sandblasted/acid-etched (SLA) and modified sandblasted/acid-etched (modSLA) Ti surfaces.

The surface properties of Ti disks are summarized in Table 1 for mean peak to valley roughness (Ra), air-water contact angle and surface chemical composition. PT Ti presented a smooth surface with a very low value of Ra. SLA and modSLA surface had similar average surface roughness, with Ra value of around 1.78 μm. PT surface showed hydrophobic with a contact angle of 109.9 ± 3.8, while modSLA surface was hydrophilic with the contact angle approximating zero (Fig. 1a). As to SLA surface, the contact angle was between the values of PT and modSLA surface. XPS analysis indicated signals of Ti, O, C and N on Ti surfaces. ModSLA surface demonstrated reduced carbon (C) concentration and increased oxygen (O) concentration (C, 32.74; O, 50.58 at %) in comparison with SLA surface (C, 39.19; O, 45.7 at %) and PT surface (C, 40.86; O, 43.88 at %).

Scanning electron microscopy (SEM) images (Fig. 1b) showed that PT surface was relatively smooth with parallel grooves and turned traces. A same topography was found in SLA and modSLA surface, both of which exhibited an irregular rough morphology with many craters and micropits.

### DCs adhesion and morphology

**Figure 2:**
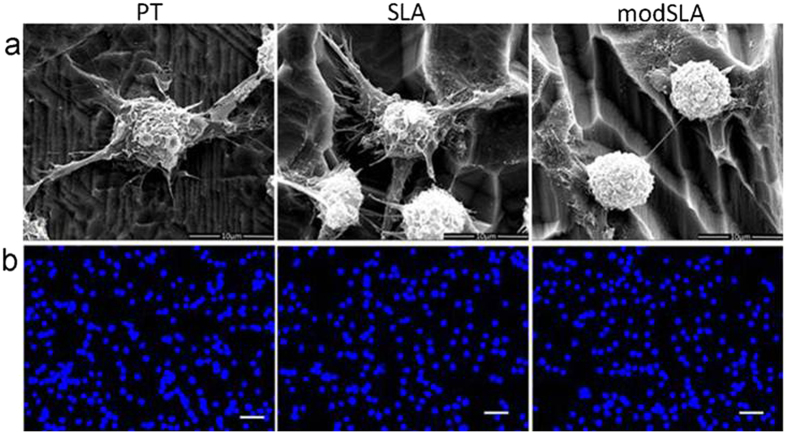
SEM micrographs and DAPI staining of DCs seeded on Ti surfaces. (**a**) SEM micrographs showed the morphologies of DCs adherent on Ti disks. High magnification micrographs (original magnification ×1000, bar = 10 μm) indicated that DCs on PT and SLA surface were irregular in morphology and exhibited multiple dendritic processes, associated with mDCs; DCs on modSLA surface possessed mostly spherical morphology with few cellular processes, similar to iDCs. (**b**) DAPI staining showed that the number of DCs adherent on different Ti surfaces was similar. (Original magnification ×200, bar = 50 μm).

SEM revealed that DCs seeded on PT and SLA surfaces exhibited obvious dendritic processes, similar to mDCs. DCs induced by modSLA were round in morphology with few processes, associated with iDCs (Fig. 2a). The number of DCs attached to three different Ti surfaces was not significantly different, which was confirmed by the images of DAPI staining (Fig. 2b).

### Expression of DC surface markers

**Figure 3:**
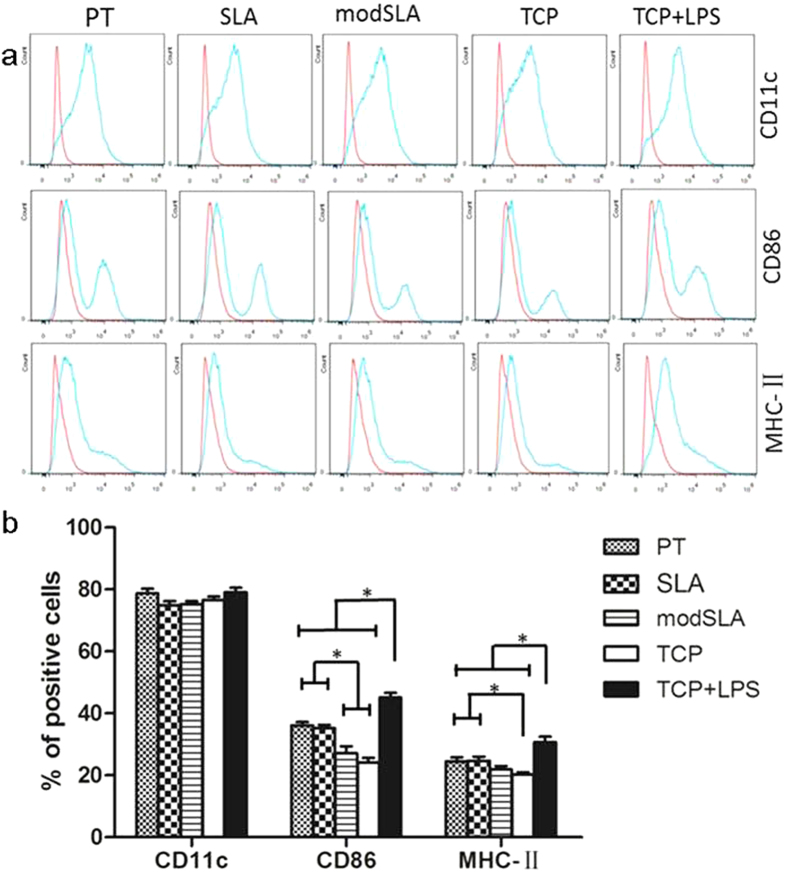
Flow cytometric analysis of surface markers by DCs treated with Ti surfaces (PT, SLA and modSLA) in comparison with iDC (TCP) and mDC (TCP + LPS). (**a**) The surface marker molecules were analyzed by flow cytometry. (**b**) Quantification of the positive cells. Data are expressed as mean ± SEM, n = 3. *p < 0.05.

As shown in Fig. 3, the expression level of CD11c, the specific surface marker of myeloid DCs, was almost up to 80% by Ti-treated DCs, as well as DCs seeded on tissue culture plate (TCP) with or without lipopolysaccharide (LPS) stimulation. As expected, LPS treatment lead to a prominent increase in the expression of CD86 and MHC-II, indicative markers of DC maturation. DCs treated with PT and SLA surface upregulated CD86 expression as compared with modSLA-treated DCs and iDC (TCP). There was no significant difference between PT- and SLA-treated DCs. This trend accompanied the expression of MHC-II. Both PT- and SLA-treated DCs expressed higher levels of MHC-II than iDCs, while the level of MHC-II expressed by modSLA-treated DCs was similar to that by iDC.

### Cytokine expression

**Figure 4:**
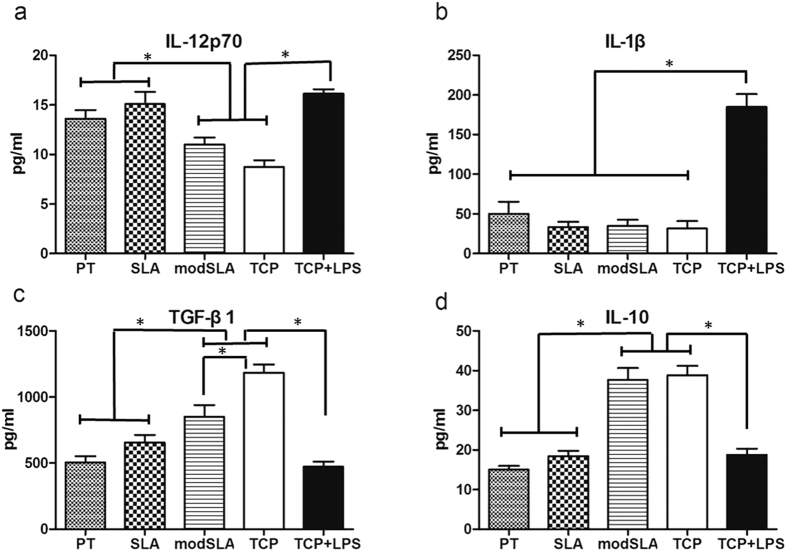
Cytokine production by DCs treated with Ti surfaces (PT, SLA or modSLA) compared with iDC (TCP) and mDC (TCP + LPS) controls. Measurements of cytokines (**a**) IL-12p70, (**b**) IL-1β, (**c**) TGF-β, (**d**) IL-10 in cell culture supernatant were performed by ELISA. Data are expressed as mean ± SEM, n = 3. *p < 0.05.

ELISA analysis showed that cytokine production was differently dependent on Ti surfaces (Fig. 4). PT- and SLA-treated DCs released higher levels of IL-12p70 in comparison with the iDC control (p < 0.05) and to a level similar to LPS-stimulated DCs. The IL-12p70 production by modSLA-treated DCs was not significantly different from iDCs. DCs induced by different Ti surfaces produced similar amounts of IL-1β. No obvious difference was found between Ti-treated DCs and iDCs. The secretion level of IL-10 by PT- and SLA-treated DCs was similar, which was not different from that of LPS-induced DCs. ModSLA surface treatment induced DCs to produce higher amounts of IL-10 compared with PT and SLA treatment (p < 0.05), similar to TCP control. The trend of TGF-β1 expression was similar to that of IL-10. The only different point was that the level of TGF-β1 by modSLA-treated DCs was significantly lower than the iDC control.

### Analysis of the osteogenic gene expression

To investigate whether the soluble factors secreted by Ti-treated DCs affect the osteogenic differentiation of preosteoblasts MC3T3-E1 cells, DC supernatant was collected as conditioned media (CM).

**Figure 5:**
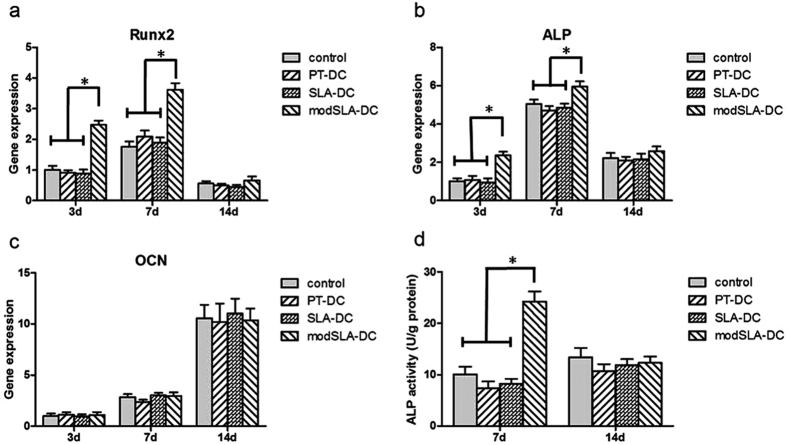
Osteogenic differentiation of MC3T3-E1 cells in the presence of osteogenic medium mixed with CM from Ti-treated DCs compared with the control group (monoculture without CM). Relative mRNA levels of (**a**) Runx2, (**b**) ALP and (**c**) OCN during cell differentiation of MC3T3-E1 cells. Ratios of target genes relative to GAPDH were expressed relative to the mean value of the control group on day 3, which was set as 100%. (**d**) ALP activity of MC3T3-E1 cells in the presence of CM from Ti-treated DCs compared with the control group after 7 and 14 days of culture. Data are expressed as mean ± SEM, n = 3. *p < 0.05.

The gene expressions of runt-related transcription factor 2 (Runx2), alkaline phosphatase (ALP) and osteocalcin (OCN) by MC3T3-E1 cells were shown in Fig. 5a–c. On day 3 of culture, CM obtained from modSLA-Ti treated DCs significantly up-regulated the mRNA levels of Runx2 and ALP relative to the control group (p < 0.05). For MC3T3-E1 cells cultured in the presence of CM from PT- and SLA-Ti treated DCs, the mRNA levels did not differ from the control group (without the addition of CM). On day 7, the expression of Runx2 and ALP gene showed an increase and the differential expression of mRNA among the groups was similar to that on day 3. The expression of OCN mRNA, a late marker of osteogenic differentiation, showed no significant difference among the groups.

### Analysis of ALP activity

The ALP activity of MC3T3-E1 cells was showed in Fig. 5d. On day 7, MC3T3-E1 cells co-cultured with CM from modSLA-induced DCs demonstrated statistically higher ALP activities than the control group (p < 0.05), whereas CM derived from PT- and SLA-induced DCs did not significantly alter ALP activity compared with the control group. On day 14, for MC3T3-E1 cells in the presence of CM from modSLA-treated DC, the ALP activity decreased and no difference was observed among the groups.

## Discussion

In a recent report, it was illustrated that the development of bone replacement biomaterials had been switched from inert to immune-modulatory^17^. Trindade *et al*. believed that this transformation in paradigm also occur in the field of dental implants^8^. Upon titanium dental implants get in contact with any living tissue, a host response is unavoidable. In the early phase of inflammation, the implant surface is recognized by the immune system. Then osteoblasts may come into contact with cells of the immune system during the process of osseointegration. DCs, potent inducer and modulator of the immune response in the immune system, play a critical role in the host response to implant biomaterials^2^. In this paper, the effect of Ti surfaces on DC phenotype and maturation was evaluated. Furthermore, CM from DCs treated with Ti was collected and co-cultured with preosteoblast MC3T3-E1 cells to unveil the cellular communication. Further understanding of how this communication takes place is significant for the future design of implant surfaces.

Based on the flow cytometry analysis, CD11c, the specific surface marker of myeloid DCs, was highly expressed in all groups, confirming that DCs can be generated with the only stimulation of high levels of GM-CSF in the absence of IL-4^4^,^18^. Co-stimulatory molecule CD86 is the most sensitive marker for DC response to biomaterial treatments and is a valid variable for determining DC maturation levels^6^. DCs induced by PT and SLA surface expressed higher levels of DC maturation markers (CD86 and MHC-II) compared with the TCP control (iDC). DCs treatment with modSLA surface did not influence MHC-II expression relative to iDCs. The levels of CD86 and MHC-II in mDC group were significantly higher than other groups, which was consistent with the previous knowledge that LPS could modulate DC maturation^19^.

Under the phase contrast microscope, iDCs were mostly spherical in morphology and exhibited few dendritic processes, while LPS-treated mature DCs increased in size and presented extensive cellular processes. SEM showed that DCs seeded on PT and SLA surface exhibited multiple dendritic processes, similar to mDCs; DCs treated with modSLA-Ti surface possessed a spherical morphology that was associated with iDCs.

In addition, release of pro-inflammatory cytokines is another indicating of DC maturation^20^. ELISA analysis showed that DCs treatment with Ti surface induced differential cytokine production. Consistent with up-regulated expression of DC maturation markers, LPS-induced DCs released significantly high level of pro-inflammatory cytokine IL-1β. Compared with modSLA-treated DCs, DCs seeded on PT and SLA surfaces expressed higher level of pro-inflammatory cytokine IL-12p70, to a level similar to mDCs. As to the anti-inflammatory cytokine IL-10 and TGF-β, DCs treatment with modSLA surface resulted in enhanced cytokine production, similar to iDCs. Above all, cytokine analysis indicated that PT and SLA surfaces tended to generate a pro-inflammatory environment, while modSLA surface was non-inflammatory for DCs.

According to the collective analysis of DCs morphology, surface marker expression and cytokine production, it was indicated that Ti disks with different surface treatments (PT, SLA and modSLA) resulted in differential DC responses. PT and SLA treatment promoted DC mature, while treatment with modSLA surface maintained an iDC phenotype, consistent with a previous study^21^.

With similar surface chemical compositions and distinct values of the surface roughness, PT and SLA surfaces induced the same DC phenotype, suggesting that the surface roughness was not the critical factor in regulating DC maturation. Conversely, modSLA and SLA surface were same in the surface roughness, but showed differences in surface chemical compositions and surface energy. These results suggested that the surface hydrophilicity and chemical property played important roles in modulating DCs response to biomaterials, which had been pointed out by several researchers^1^,^5^. Surface wettability influences the degree of contact between the implant surface and the biologic environment^22^. On hydrophilic implant surfaces, molecules are adsorbed in a more flexible pattern, leading to an enhanced interaction between the implant surface and the surrounding environment. Previous studies reported that hydrophilic surfaces demonstrated anti-inflammatory property by mitigating the pro-inflammatory cytokines production and inflammatory response of macrophages^23^,^24^. Since surface chemistry influences surface wettability^22^, the surface chemistry may be the most critical factor in modulating DC response. Buser and colleagues reported that the oxide surface is hydrophilic^14^. In the present study, increased content of O element was detected on modSLA surface, which promoted an iDC phenotype, consistent with a previous study^21^.

Preosteoblast MC3T3-E1 cells undergo a developmental sequence of proliferation and differentiation similar to the primary cells in culture^25^,^26^. Osteoblast differentiation and maturation are characterized with the changes in expression of a series of osteogenic-specific genes at each developmental stage^27^. Runx2, a master regulator of osteogenic gene expression, is necessary for the osteoblast lineage commitment and modulates osteoblast differentiation^28^. The expression of ALP, an early marker of osteogenic differentiation, up-regulates with the maturation of extracellular matrix and down-regulates with the mineralization process^29^. Furthermore, ALP activity has a positive relation with osteoblast differentiation and maturation. OCN, a late marker of osteogenic differentiation, begins to express when the ALP expression reaches its peak and is associated with the mineralization of osteoblasts^30^,^31^. RT-PCR results showed that osteoblasts cultured in the presence of CM from modSLA-induced DCs demonstrated statistically higher levels of Runx2 and ALP mRNA compared with the control group. ALP activity analysis further demonstrated the gene expression status among the different groups. In addition, the level of ALP activity achieved its peak on day 7 and then decreased slightly after 14 days, in accordance with the previous study result^9^. Based on the analysis of gene expression and ALP activity, it appeared that iDCs treated with modSLA surface promoted the early stage of osteogenic differentiation of MC3T3-E1 cells through certain soluble factor, while PT and SLA-treated DCs had no effect on osteoblast activity.

Dental implant implantation is always accompanied with injury through the surgical procedure. Injury to the tissue initiates an inflammatory response to the implanted biomaterial^1^. It was pointed out that manipulating the potency of inflammatory response would be novel therapeutic approaches to enhance osteogenesis^32^. The results herein imply that the hydrophilic modSLA surface may lead to an anti-inflammatory environment by maintaining an iDC phenotype, which exhibited a potential of promoting the early osteogenic differentiation of MC3T3-E1 cells. Our findings provide knowledge to understand the accelerated osseointegration of Ti implants with modSLA surface in the level of cellular communication. As to the application of dental implant, induction of iDC at the implant site would provide a meaningful strategy to restrict the immune response and to expedite wound healing and osseointegration.

## Materials and Methods

### Titanium disks

The Grade 2 unalloyed Ti disks (1 mm in thickness and 14 mm in diameter) were supplied by National Engineering Research Center of Biomaterials, Sichuan University, China. The surfaces of PT, SLA and modSLA were processed with previously described methods^15^. PT substrates were polished to have a smooth Ti surface. SLA surfaces were produced by sandblasting and acid etching of PT surfaces. ModSLA surfaces were prepared with the same treatments as SLA but were rinsed under nitrogen protection to prevent exposure to air, and then directly stored in an isotonic NaCl solution. The disks were sterilized by gamma irradiation over night before using.

The surface roughness of Ti disks was measured using a mechanical profilometer (Perthometer M1, Mahr, Germany) and the surface wettability was determined by dynamic contact angle measurements (DSA30, Kruss, Germany). The chemical composition of Ti surfaces was analyzed by X-ray photoelectron spectroscopy (XPS) (AXIS Ultra DLD, Kratos Cop. England). The surface morphology of the disks was observed under SEM (Inspect F, FEI, USA).

### Generation of murine bone marrow-derived DC

Male C57BL/6 mice aged 6–8 week old were used in this study and maintained in accordance with the guidelines of the Care and Use of Laboratory Animals published by the China National Institute of Health. The protocol was approved by the Ethics Committee of West China College of Stomatology, Sichuan University (Permit Number: SKLODLL2013A073).

DCs were prepared from mouse bone marrow using an established protocol^33^ with some modifications. Briefly, bone marrow cells were flushed from femur and tibia. Erythrocytes were lysed with RBC lysis buffer. Then, the harvested cells were cultured in RPMI-1640 complete medium with 10% fetal bovine serum (FBS) (Gibco, Grand Island, NY), 1% penicillin/streptomycin and 20 ng/ml rmGM-CSF (PeproTech, Rocky Hill, NJ). At day 3, fresh medium containing GM-CSF was added to each culture flask. At day 6, half of the cell suspension was centrifuged, resuspended in the same volume of cell culture medium and returned to the culture flask. After incubation for another two days, the cells were harvested and used for experiments.

### DCs treatment

On day 8 of culture, loosely adherent and non-adherent cells were collected and seeded on Ti disks in 24-well plates at a density of 5 × 10^5^ cells/ml. Cells treated with 1000 ng/ml LPS (Sigma) were used as the positive control of mDCs, while cells without any treatment in TCP were used as the negative control of iDCs. After 24 h of differential treatments, DCs were harvested for analysis.

### Scanning electron microscopy of DCs

After 24 h exposure to Ti disks, the cells were washed with PBS, fixed with 2.5% glutaraldehyde at 4 °C overnight, and dehydrated with a graded series of ethanol (30%, 50%, 75%, 85%, 95% and 100%) for 15 min at each concentration. Then, the samples were permuted with isoamyl acetate for 5 min and dried overnight at room temperature. Subsequently, specimens were sputter-coated with a thin layer of gold. The micrographs were collected using a scanning electron microscope (Inspect F, FEI).

### Flow cytometry of DC surface markers

After 24 h of cell culture, DCs from the five groups were harvested for the anlysis of surface markers expression. The cells were resuspended in PBS containing 1% bovine serum albumin and stained with fluorescently labeled antibodies, including PE-anti-CD11c, FITC-anti-CD86 and Alexa Fluor-anti-MHC-II (all from BD Biosciences, USA) for 30 minutes at 4 °C. Unstained controls were used for the establishment of background fluorescence. The stained cells were analyzed by a flow cytometer (Cytomics TM FC500; Beckman, USA). Data analysis was performed with FlowJo7.6 (Tree Star, Ashland, OR).

### DAPI staining

The numbers of DCs attached to Ti surfaces were quantified using DAPI staining assay. DCs on Ti disks were washed twice with PBS and fixed in a 4% formaldehyde solution for 30 min at room temperature. Subsequently, the samples were washed again with PBS and mounted with DAPI medium at a final concentration of 100 ng/ml. The samples were analyzed by fluorescence microscope (Olympus IX71-F22FL, Japan) and the nuclei were counted using the ImageJ software.

### Cytokine measurements

As a complementary method of evaluating DC responses to different Ti surfaces, measurements of pro-inflammatory cytokines (IL-1β, IL-12p70) and anti-inflammatory cytokines (IL-10, TGF-β1) in DC culture supernatants were performed with ELISA kits (Boshide, Wuhan, China) according to the manufacturer’s instructions.

### Conditioned media and culture of MC3T3-E1 cells

To investigate whether the soluble factors secreted by Ti-treated DCs affect the osteogenic differentiation of preosteoblasts, CM were collected by centrifugation of the cell supernatant and stored at −80 °C prior to use.

The murine preosteoblast MC3T3-E1 cells were purchased from the cell bank of Chinese academy of sciences and incubated in osteogenic medium (100 nM dexamethasone, 10 mM sodium β-glycerophosphate and 50 μg/ml ascorbic acid) mixed with CM at the 1:1 ratio. Culture medium containing CM was replaced every three days. The monoculture of MC3T3-E1 cells was used as the control group. Cultures were maintained at 37 °C in a humidified atmosphere of 5% CO_2_.

### Quantitative RT-PCR

MC3T3-E1 cells were seeded at a density of 1 × 10^4^/ml. After 3, 7 and 14 days of culture, total RNA was extracted from MC3T3-E1 cells using TRIZOL reagent (Invitrogen, USA) and reverse transcribed to cDNA using QuantiTec reverse transcription kit (Qiagen, Valencia, CA, USA)^34^. For quantitative PCR, 2 μl cDNA, 0.8 μl of the sense and antisense specific primers and 10 μl SYBR Premix Ex TaqTM were used in a final reaction volume of 20 μl. The amplification program was as following: a preincubation step (30 s 95 °C), followed by 40 cycles consisting of a denaturation step (5 s 95 °C), an annealing step (31 s 60 °C), and an extension step (30 s 72 °C). Three target genes were investigated, namely: Runx2, ALP and OCN. Glyceraldehyde-3-phosphate dehydrogenase (GAPDH) was used as the housekeeping gene. Details of the primers are shown in Table S2. The mRNA levels of the target genes were calculated, normalized to GAPDH according to the formula 2^−ΔΔCt^ and expressed as fold increase relative to the expression of the control group at day 3 (=1).

### ALP activity assay

ALP activity was measured as described previously^35^. Briefly, on 7 and 14 days, cells were washed twice with ice-cold PBS and digested by trypsin. After washes by centrifugation, the cells were lysed with freezing-thawing and sonication on ice for three times. ALP activity was measured using ALP activity kit (Nanjing Jiancheng, China). The absorbance of the stopped reaction was read at 405 nm. Aliquots of supernatants were subjected to total protein assay, which was measured by bicinchoninic acid protein measurement kit (KeyGen Biotech, China).

### Statistical analysis

Data are expressed as mean ± SEM. Statistical analysis was performed by the one-way ANOVA followed by Student-Newman-Keuls *post-hoc* test. Results were considered statistically significant at the p < 0.05 level.

## Additional Information

**How to cite this article**: Zheng, X. *et al*. Effect of Different Titanium Surfaces on Maturation of Murine Bone Marrow-Derived Dendritic Cells. *Sci. Rep.* **7**, 41945; doi: 10.1038/srep41945 (2017).

**Publisher's note:** Springer Nature remains neutral with regard to jurisdictional claims in published maps and institutional affiliations.

## Supplementary Material

Supplementary Table S2

## Figures and Tables

**Table 1:** The surface characteristics of Ti disks with different surface treatments.

n = 5	PT	SLA	ModSLA
Roughness (Ra) (μm)	0.38 ± 0.02*	1.78 ± 0.02	1.78 ± 0.04
Contact angle (θ)	109.9 ± 4.17*	77.4 ± 3.49*	10.1 ± 0.43*
Surface chemical composition			
O (%)	43.88 ± 1.13	45. 7 ± 1.12	50.58 ± 2.03*
Ti (%)	13.48 ± 0.49	13.75 ± 1.84	15.17 ± 0.56
N (%)	1.78 ± 0.14	1.37 ± 0.13	1.51 ± 0.14
C (%)	40.86 ± 1.47	39.19 ± 1.95	32.74 ± 1.60*
