# Supplementary material for: Effect of Different Titanium Surfaces on Maturation of Murine Bone Marrow-Derived Dendritic Cells
Source: Sci Rep. 2017 Feb 3;7:41945. doi: 10.1038/srep41945 (PMC5291203; doi:10.1038/srep41945)
Supplement: Supplementary Table S2 [file srep41945-s1.pdf]

Effect of Different Titanium Surfaces on Maturation of Murine Bone Marrow-Derived Dendritic Cells

Xiaofei Zheng[1,2], Fengjuan Zhou[1,3], Yifei Gu[1,2], Xiaobo Duan[1,2], Anchun Mo[1,2*]

[1] State Key Laboratory of Oral Diseases, West China Hospital of Stomatology, Sichuan University, Chengdu, Sichuan, China

[2] Dental Implant Center, West China Hospital of Stomatology, Sichuan University, Chengdu, Sichuan, China

[3] Geriatric Dentistry Department, West China Hospital of Stomatology, Sichuan University, Chengdu, Sichuan, China

Table S2. Sense (+) and antisense (-) of the primers used in the real-time PCR of target and housekeeping genes

Gene	GeneBank accession number	Primer sequence(5'-3')	Amplicon size (bp)
GAPDH	NM_008084.2	(+) 5'-GACATCAAGAAGGTGGTGAAGC-3'<br>(-) 5'-GAAGGTGGAAGAGTGGGAGTT-3'	117bp
Runx2	NM_009820.4	(+) 5'-GCCGGGAATGATGAGAACTA-3'<br>(-) 5'-GGACCGTCCACTGTCACTTT-3'	200bp
ALP	NM_007431.2	(+) 5'-AACCCAGACACAAGCATTCC-3'<br>(-) 5'-GCCTTTGAGGTTTTTGGTCA-3'	200bp
OCN	NM_031368.4	(+) 5'-TTGGTGCACACCTAGCAGAC-3'<br>(-) 5'-ACCTTATTGCCCTCCTGCTT-3'	151bp
